# Extension of Lecture Terms in Medical Colleges

**Published:** 1848-07

**Authors:** 


*Extension of Lecture Terms in Medical Colleges.*—We perceive by our exchanges, and by circulars, that several colleges have extended the term of lectures. The Jefferson College, of Philadelphia, which was supposed to regard unfavorably the project of extension, announces that the next course will continue nearly twenty weeks. The Georgia Medical College has extended to five months, and so has the Louisville College. The Medical Department St. Louis University has added two weeks. We believe the list to be larger, but others do not at the moment occur to us. We doubt not the improvement will soon be generally adopted.

